# Bacterial community in homemade kimchi and probiotic characterization of *Lactiplantibacillus plantarum* HQ04

**DOI:** 10.3389/fmicb.2025.1700442

**Published:** 2025-11-28

**Authors:** Wei Zhao, Meilin Chen, Yixuan Zhang, Yiping Fan, Pinyao Zhao, Ling Zhou, Weimin Ouyang, Jie Wang

**Affiliations:** 1 Solid-State Fermentation Resource Utilization Key Laboratory of Sichuan Province, Yibin University, Yibin, Sichuan, China; 2 Faculty of Quality Management and Inspection Quarantine, Yibin University, Yibin, Sichuan, China; 3 Yibin Municipal Bureau of Agriculture and Rural Affairs, Yibin, Sichuan, China

**Keywords:** homemade fermented kimchi, *Lactiplantibacillus plantarum*, probiotic properties, bacteriocin, class II bacteriocin gene cluster

## Abstract

Homemade fermented kimchi is rich in microbial communities and is regarded as an important source of probiotics. In this study, the bacterial community diversity of 40 kimchi samples from Yibin City, China, was analyzed using high-throughput sequencing of 16S rRNA gene. Then, *Lactiplantibacillus plantarum* (*L. plantarum*) strains were isolated from the kimchi samples and characterized for their probiotic properties. The strain with the most favorable probiotic traits was further analyzed to identify its genetic characteristics through whole genome sequencing. The results showed that Firmicutes (57.9%) and Proteobacteria (36.3%) were the dominant phyla, whereas *Levilactobacillus* (19.2%) and *Lactiplantibacillus* (12.2%) were the most abundant genera. Alpha- and beta-diversity analyses indicated that each kimchi sample comprised an independent microbial ecosystem. A total of 11 *L. plantarum* strains were isolated, among which strain HQ04 demonstrated superior probiotic properties, such as high tolerance to acid and bile salts, gamma-aminobutyric acid (GABA) production, strong autoaggregation and coaggregation abilities, and potent antibacterial activity. Whole genome sequencing identified five secondary metabolite gene clusters in the HQ04 genome. Additionally, a class II bacteriocin gene cluster encoding Bacteriocin IIc, Enterocin X chain beta, and Plantaricin E/F was identified. These findings highlight the rich microbial diversity of homemade kimchi from Yibin and suggest that the HQ04 strain is a potential probiotic candidate for controlling pathogenic bacteria.

## 1 Introduction

Fermented foods are an integral part of the human diet worldwide because large quantities of nutritious and healthy foods that have unique flavors and health benefits are produced and preserved in this manner. The southern region of Sichuan Province in China is nourished by the Yangtze River system and has a rich history of numerous cultivated fermented foods, such as Baijiu (wine), yacai (sprouted vegetables), kimchi (fermented vegetables), Douchi (fermented black beans), and suancai (sour vegetables). Among these, kimchi is a multifunctional food with high nutritional value and distinctive flavors. It is considered one of the most widespread traditional pickled foods globally (Behera et al., 2020; Tharmabalan et al., 2025). Beyond its culinary appeal, kimchi contains abundant physiologically active compounds that possess health-promoting biological functions, such as regulation of the intestinal microecological environment, reduction of cholesterol, blood pressure, and blood lipid levels, inhibition of tumor activity, and enhancement of immune function (Song et al., 2023; Fijan et al., 2024). The quality and health benefits of kimchi are intrinsically linked to its diverse microbial communities, which are dominated by lactic acid bacteria (LAB), yeasts, molds, and other bacteria (Song et al., 2023; Pyo et al., 2024). Notably, several fermented kimchi products on the market are homemade, and unlike commercially produced kimchi, the microbial communities in homemade kimchi are highly influenced by local environmental conditions and family traditions (Lee et al., 2017; Thierry et al., 2023). These factors have led to variable microbial communities in kimchi, which further indicates that each homemade fermented kimchi can be regarded as an independent bacterial ecosystem (Lee et al., 2017; Patra et al., 2016). Thus, the unique and variable microbial population in kimchi could be an important source for screening novel probiotics.

*Lactiplantibacillus plantarum* is the most functionally significant microbial group that plays a pivotal role in kimchi fermentation (Lin et al., 2025). *L. plantarum* is a beneficial bacterium that is widely distributed in nature and is renowned for its excellent probiotic properties and safety (Zhang et al., 2023). Strains of this species colonize a variety of environments, including food, water, soil, and feces (Fidanza et al., 2021; Filannino et al., 2018). Many *L. plantarum* strains exhibit probiotic properties, such as antibacterial activity, antioxidant activity, dyslipidemia regulation, intestinal inflammation improvement, intestinal homeostasis modulation, and immunomodulation. These properties contribute significantly to the improvement of human health (Huidrom et al., 2024; Liu et al., 2022; Zhou et al., 2021). In particular, the antimicrobial functionality of *L. plantarum* has recently gained increasing attention, as many *L. plantarum* strains produce various antimicrobial compounds, including organic acids, hydrogen peroxide, bacteriocin, and other metabolites that effectively combat multiple pathogens (Huidrom et al., 2024; Wang et al., 2023; Chen et al., 2024). Bacteriocins are ribosomally synthesized bioactive peptides or proteins that are released into the extracellular space. They exhibit potent bactericidal and bacteriostatic activities and are non-toxic, harmless, non-resistant, non-residual, and stable (Heilbronner et al., 2021). Consequently, they are used as biological food preservatives and could be ideal alternatives to antibiotics and chemical preservatives owing to their superior antimicrobial activity (Isaac et al., 2025). Furthermore, bacteriocin production is an important trait of probiotics in actual food production scenarios. In fact, >99% of bacteria produce bacteriocins; however, most of these have not yet been identified (Yang et al., 2014). Furthermore, bacteriocinogeny appears to be highly strain-specific (Jiang et al., 2023). Hence, the characteristics and biological effects of individual probiotics must be analyzed and evaluated at the genetic level.

To the best of our knowledge, microbial resources in homemade fermented kimchi from Yibin City have rarely been investigated. Specifically, bacteriocin-producing *L. plantarum* strains isolated from homemade fermented kimchi in Yibin have not yet been isolated or characterized. Therefore, the objectives of this study are (1) to elucidate the diversity of the bacterial communities in 40 homemade kimchi samples from 10 districts in Yibin City, (2) to isolate, identify, and analyze the antibacterial activity of *L. plantarum* strains in homemade kimchi samples, and (3) to perform genomic analysis of the most promising candidate probiotic strains. We believe that these results will improve our understanding of bacterial diversity in homemade kimchi from Yibin and highlight potential probiotic strains for applications in the food industry.

## 2 Materials and methods

### 2.1 Bacterial diversity analysis

#### 2.1.1 Collection of the samples

In total, 40 homemade kimchi samples were collected from 10 districts (Cuiping, Xuzhou, Pingshan, Nanxi, Jiangan, Changning, Gongxian, Gaoxian, Junlian, and Xingwen) in Yibin City, Sichuan Province, China (Supplementary Figure S1). Based on the geographical locations of the 10 districts, the 40 samples were divided into 10 groups: CP, XZ, PS, NX, JA, CN, GO, GA, JL, and XW. Each group consisted of four samples. Notably, these kimchi samples were collected from citizens who manufactured homemade kimchi for personal consumption and allowed natural fermentation without the addition of commercialized probiotics. The vegetables used and salt concentrations were not restricted. All kimchi samples were loaded into 50 mL sterile Eppendorf tubes and immediately transported to the laboratory in an ice box. Each sample was divided into two parts. One part was stored at −80 °C for high-throughput sequencing analysis. The second part was stored at 4 °C for bacterial isolation and identification.

#### 2.1.2 DNA extraction and 16S rRNA gene sequencing

Microbial DNA was extracted from 40 kimchi juice samples using the QIAamp DNA Stool Mini Kit (QIAGEN, Germany) following the manufacturer’s protocols. The purity and concentration of the extracted DNA were verified using a 2% agarose gel (Invitrogen, United States) and a NanoDrop spectrophotometer (Thermo Fisher Scientific, Waltham, MA, United States), respectively. High-throughput Illumina sequencing of the V3–V4 hypervariable regions of the bacterial 16S rRNA gene was performed after amplification using the universal primer sequences 338F (5′-ACTCCTACGGGAGGCAGCA-3′) and 806R (5′-GGACTACHVGGGTWTCTAAT-3′). PCR was performed using 12.25 μL of Phusion High-Fidelity PCR Master Mix (New England Biolabs), 10 pM of forward and reverse primers, and approximately 10 ng of template DNA. Thermal cycling consisted of an initial denaturation at 98 °C for 5 min; 25 cycles of denaturation at 98 °C for 30 s, annealing at 53 °C for 30 s, and elongation at 72 °C for 45 s; and a final extension for 5 min at 72 °C. All amplified PCR products were extracted from a 2% agarose gel and purified using the AxyPrep DNA Gel Extraction Kit (Axygen Biosciences, Union City, CA, United States).

#### 2.1.3 Bacterial community analysis

FASTQ files were processed in QIIME2 version (v) 2021.4,^1^ using the DADA2 package (v 1.2) to cluster the reads into amplicon sequence variants (ASVs). Low-abundance ASVs (<0.001% of the total abundance) were filtered using the Greengenes database (Release 13.8, http://greengenes.microbio.me/). Then, the ASVs were taxonomically annotated against the SILVA database version 138.1 (doi: 10.5281/zenodo.4587955) using the Mothur software (v1.39.5). Community richness and diversity were determined using the QIIME diversity core-metrics-phylogenetic command for alpha and beta diversity analyses in the QIIME2 package and the vegan (v2.6-6.1) and ggtree (v3.12.0) packages in R. Species richness (Chao1 index) and evenness (Shannon index) were calculated using Welch’s one-way ANOVA. Beta diversity was assessed using the Bray–Curtis dissimilarity metric to generate distance matrices among samples, and non-metric multidimensional scaling (NMDS) was performed in R to visualize differences and similarities in bacterial composition between the different sampling locations.

### 2.2 Isolation and identification of LAB

Based on the results of the bacterial diversity analysis, the potential *L. plantarum* was isolated from the kimchi sample. Kimchi juice was blended in phosphate-buffered saline, shaken well, and diluted to different concentrations. Then, 100 μL of each dilution was plated onto deMan–Rogosa–Sharpe (MRS) medium for incubation at 37 °C under aerobic conditions. The diversity of the collected and purified isolates was determined based on the visual aspect of the colonies. Finally, the isolated strains were stored in 50% glycerol at −20 °C for further study.

All isolates were identified using Gram staining and 16S rRNA sequence analysis. Single bacterial colonies were selected as templates for PCR and subjected to amplification using the Master Mix, according to the manufacturer’s protocol. Universal primers (27F, 5′-AGAGTTTGATCCTGGCTCAG-3′, 1492R, 5′-TACGGTTACCTTGTTACGACTT-3′) for 16S rRNA sequencing of prokaryotes were selected, and PCR was performed as previously described (Zhao et al., 2017). PCR products were electrophoresed on 1% agarose gels and sequenced by Youkang Biotech (Hangzhou Youkang Biotechnology Co., Ltd., Hangzhou, China). The closest known relatives of each isolate were determined using the BLAST program on the NCBI website.

### 2.3 Assessment of probiotic properties

#### 2.3.1 Bile salt and acid tolerance assay

The probiotic potential of the isolated strains was evaluated by performing bile salt and acid tolerance assays according to Wang et al. (2023). Briefly, bile salt tolerance was assessed by culturing each strain in MRS broth medium containing 0.1, 0.2, and 0.3% (w/v) bile salt, followed by incubation at 37 °C for 4 h. For acid resistance tests, the isolated strains were added to MRS broth medium with different pH values (pH 2.0, 3.0, and 4.0), and cultured at 37 °C for 4 h. After incubation, 100 μL of each bacterial suspension was removed and spread onto MRS solid plates for cell number estimation using the serial dilution method, followed by an inverted incubation at 37 °C for 24 h. Finally, the ratio of viable cells to the control culture was calculated. A bacterial suspension in MRS broth without acid or bile salts was used as the control.

#### 2.3.2 GABA production

GABA concentrations in the isolated strains were determined using the Berthelot colorimetric method described by Wang et al. (2023). The activated isolated strains were sequentially inoculated at 2% (v/v) inoculum concentration into glucose–yeast extract–peptone (GYP) seed medium and GYP fermentation medium, followed by incubation at 37 °C until a stable phase was achieved. Next, centrifugation (8,000 × g, 4 °C, 10 min) was performed, and 500 μL of the supernatant was added to a mixture of 200 μL borate buffer (0.2 mol/L, pH 9.0), 1 mL phenol (6%, w/v), and 400 μL sodium hypochlorite (5.5%, w/v). After violent oscillations, the mixture was heated in a boiling water bath for 10 min and then transferred to an ice bath for 20 min. After the solution turned blue, 2 mL of 60% ethanol was added and mixed. Next, OD_645_ was measured. A standard curve was constructed using GABA standards at 0, 0.2, 0.4, 0.6, 0.8, and 1.0 g/L concentrations.

#### 2.3.3 Autoaggregation and coaggregation ability

Autoaggregation and coaggregation abilities were assessed according to the method described by Wang et al. (2023). For the autoaggregation assay, 4 mL of the isolated strain suspensions (OD_600_ = 0.6 ± 0.05, A_0_) were added to a centrifuge tube, vortexed for 10 s, and incubated at 37 °C for 5 h. After incubation, the OD_600_ of the upper suspension was measured and is denoted as (A). The autoaggregation percentage (%) was calculated as (1 − A/A_0_) × 100. For the coaggregation assay, 2 mL of isolated strain suspensions (OD_600_ = 0.6 ± 0.05, A_X_) were added to 2 mL of two pathogen suspensions [*Escherichia coli* (*E. coli*) CVCC196 and *Staphylococcus aureus* (*S. aureus*) BJ216 strains] (OD_600_ = 0.6 ± 0.05, A_Y_) in centrifuge tubes, which were vortexed for 10 s and incubated at 37 °C for 5 h. After incubation, the OD_600_ of the upper suspension was measured and denoted as (A_X + Y_). The coaggregation percentage (%) was calculated as follows: (1–2 A_X + Y_/(A_X_ + A_Y_)) × 100.

#### 2.3.4 Antibacterial activity

The antagonistic activities of the kimchi isolate strains against *E. coli* CVCC196 and *S. aureus* BJ216 were evaluated using an agar plug diffusion test. Briefly, overnight cultures of *E. coli* CVCC196 and *S. aureus* BJ216 were diluted to 1 × 10^7^ CFU mL^−1^ and uniformly spread using a sterile cotton swab on Luria-Bertani (LB) agar plates. Sterile Oxford cups (6 mm) were placed on the surface of the LB agar plates. The isolated strain cultures were centrifuged, and the supernatants were filtered using a 0.22 μm syringe filter. Then, 200 μL of cell-free supernatant (CFS) was added to the Oxford cups, followed by 24 h incubation at 37 °C. Next, the diameter of the inhibitory zone around the Oxford cup was measured (including the well diameter).

The sensitivity of CFS to hydrolytic enzymes and pH was determined after treatment with trypsin and 1 mol/mL NaOH, respectively. Trypsin was diluted, filter-sterilized, added to the CFS, and incubated. Then, the enzymes in the CFS were heat-inactivated. The pH was measured, and 1 mol/mL NaOH was added to the CFS until the solution pH reached 7. Finally, antimicrobial activity was monitored as described earlier. Appropriate negative controls (sterile CFS) were used in the assays.

### 2.4 Whole genome sequencing and genome mining of selected strain

The total genome of the selected strain, which had excellent antimicrobial properties, was sequenced for further characterization. The bacterial cells were sent to Shanghai Personal Biotechnology Co., Ltd. (Shanghai, China) for whole genome sequencing. Total DNA was extracted using the QIAamp DNA Microbiome Kit (QIAGEN, Hilden, Germany). Concentration, purity, and integrity assessments were performed using a Qubit 4.0 fluorometer (Thermo Fisher Scientific, United States), a NanoDrop^®^ ND-1000 Spectrophotometer (Thermo Fisher Scientific, United States), and agarose gel electrophoresis, respectively. The second and third generations of complete genome sequencing were performed using the Illumina NovaSeq PE150 and Oxford Nanopore ONT sequencing platforms, respectively. Next, we constructed a genomic library for second- and third-generation sequencing using the TruSeqTM DNA Sample Prep Kit and SQK-LSK109 connection kit, respectively. Unicycle (v 0.5.0) and Flye (v 2.9.1) software were used to assemble the third-generation sequencing data, and Pilon (v 1.24) was used to correct the sequencing data. Finally, the contigs were spliced to obtain complete sequences.

Genomic features were annotated using the NCBI Prokaryotic Genome Annotation Pipeline (PGAP). CGView software was used to draw chromosomal and plasmid genome circle diagrams. The plasmids and selected chromosomal sequence regions were subjected to homology searches using BLAST. Secondary metabolite and prebiotic biosynthesis gene cluster analyses were performed on the genomic data of the isolated strains using the antiSMASH bacterial version,^2^ and putative bacteriocin-encoding genes were identified in the genome using BAGEL4.^3^ Candidate genes related to probiotic properties in the genome were mined based on the annotation results.

### 2.5 Statistical analysis

All experiments were performed with at least three independent replicates (*n* ≥ 3). The results are presented as mean ± standard deviation (SD). Differences between various treatments were analyzed by one-way analysis of variance (ANOVA) and Duncan’s multiple comparison tests using the SPSS 22.0 statistical package (SPSS Inc., Chicago, United States). The *p*-value representation was as follows: “*” for < 0.05 and “**” for < 0.01.

## 3 Results

### 3.1 Bacterial diversity and community structure of kimchi samples

**Figure 1:**
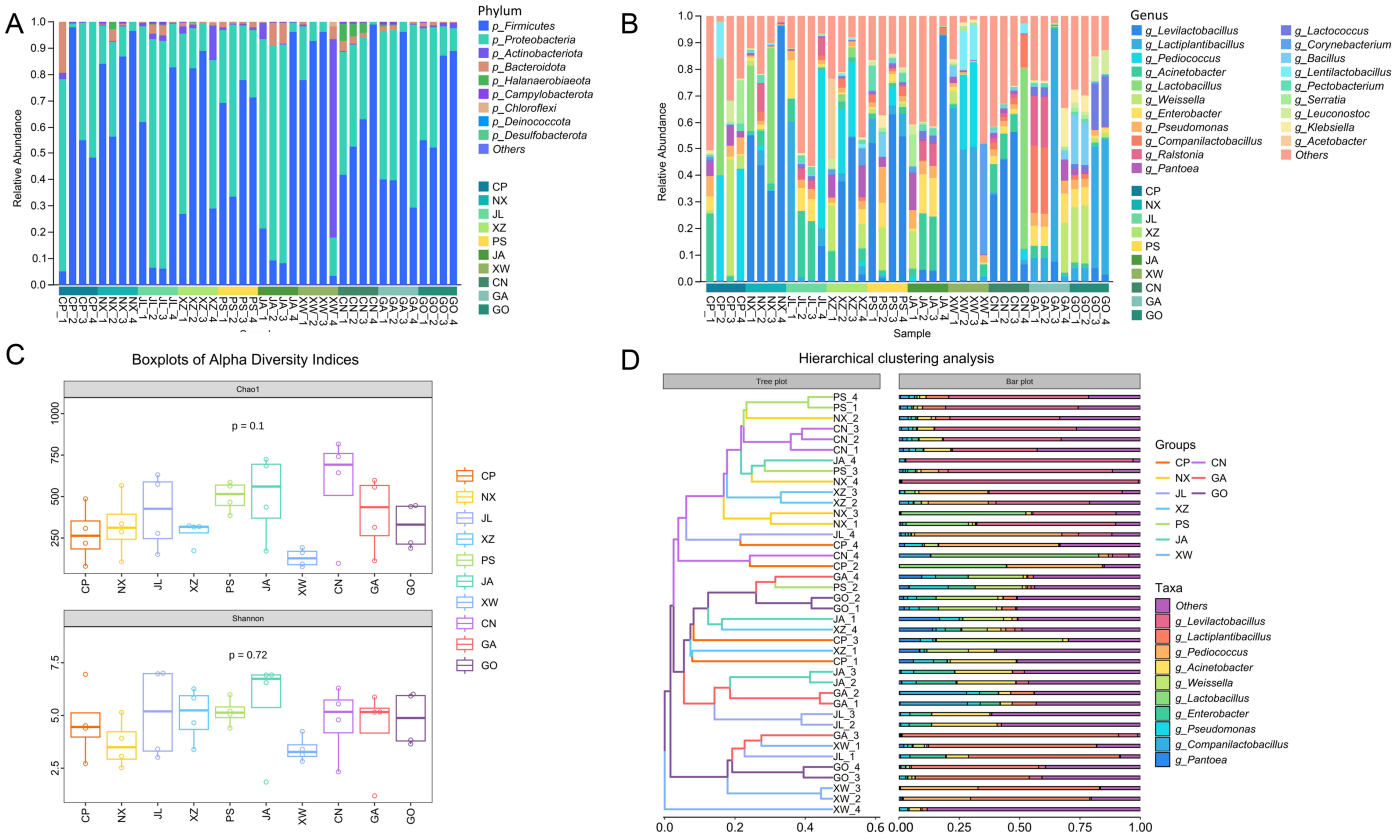
Bacterial diversity, community structure, and taxonomic composition of the 40 kimchi samples. **(A)** Taxonomic composition at the phylum level. **(B)** Twenty most abundant genera. **(C)** Alpha diversity. **(D)** Hierarchical clustering analysis. CP, XZ, PS, NX, JA, CN, GO, GA, JL, and XW indicate the Cuiping, Xuzhou, Pingshan, Nanxi, Jiangan, Changning, Gongxian, Gaoxian, Junlian, and Xingwen districts, respectively. Differences in alpha diversity indices (Chao1 and Shannon) among the 10 districts were assessed using Welch’s one-way ANOVA. Beta diversity based on Bray–Curtis distances was visualized using non-metric multidimensional scaling.

In this study, high-throughput sequencing of the 16S rRNA gene was performed to characterize the bacterial communities in kimchi samples. This study is the first comprehensive analysis of the microbial community in homemade fermented kimchi from Yibin City, a region renowned for its diverse traditional fermented foods. The relative abundances of the top nine phyla are shown in Figure 1A. Firmicutes was the predominant phylum, with an abundance of 57.9%, followed by Proteobacteria (36.3%), Actinobacteria (3.0%), and Bacteroidetes (2.0%). At the order level, Lactobacillales was the most dominant, with a median abundance of 56%, followed by Enterobacterales (20%), Pseudomonadales (9.7%), Burkholderiales (3.7%), and Flavobacteriales (1.6%). At the genus level, *Levilactobacillus* (19.2%) and *Lactiplantibacillus* (12.2%) were the two most abundant genera, followed by *Pediococcus* (6.8%) and *Lactobacillus* (5.0%) (Figure 1B). Additionally, genera containing potential pathogens, such as *Acinetobacter*, *Pseudomonas*, *Klebsiella*, and *Serratia*, were detected in some samples.

The alpha diversity based on the Chao1 and Shannon indices ranged from 77.25 to 816.26 (average 371.89) and 1.19 to 7.00 (average 4.65), respectively (Figure 1C). No significant differences were observed in bacterial community richness or evenness among the 10 districts (Chao1: *p* = 0.1; Shannon: *p* = 0.72, ANOVA). Beta diversity analysis showed that the microbial communities did not cluster according to the geographical district of origin (Figure 1D).

### 3.2 Isolation and identification of *Lactiplantibacillus plantarum* strains

Based on the results of the bacterial diversity analysis and the list of strains that can be used in food in China, we focused on *L. plantarum* strains in subsequent experiments. In this study, 11 *L. plantarum* strains were isolated from kimchi samples based on their typical morphological characteristics (small pinpointed and creamy white colonies, Gram-positive and rod-shaped characteristics) and 16S rRNA sequence analysis (Supplementary Figure S2), namely, *L. plantarum* HQ01, *L. plantarum* HQ02, *L. plantarum* HQ03, *L. plantarum* HQ04, *L. plantarum* HQ05, *L. plantarum* HQ06, *L. plantarum* HQ07, *L. plantarum* HQ09, *L. plantarum* HQ12, *L. plantarum* HQ13, and *L. plantarum* HQ16.

### 3.3 Assessment of probiotic properties

#### 3.3.1 Tolerance to acid and bile salt

The viabilities of the 11 *L. plantarum* strains after exposure to acid and bile salt are shown in Supplementary Table S1. Except for strains HQ02, HQ09, HQ13, and HQ16, the survival rates of the other seven *L. plantarum* strains were >50% after 2 h of incubation at pH 2, 3, and 4. In 0.1, 0.2, and 0.3% bile salts, the survival rates of strains HQ04 and HQ06 were higher than those of other *L. plantarum* strains.

#### 3.3.2 GABA production

**Figure 2:**
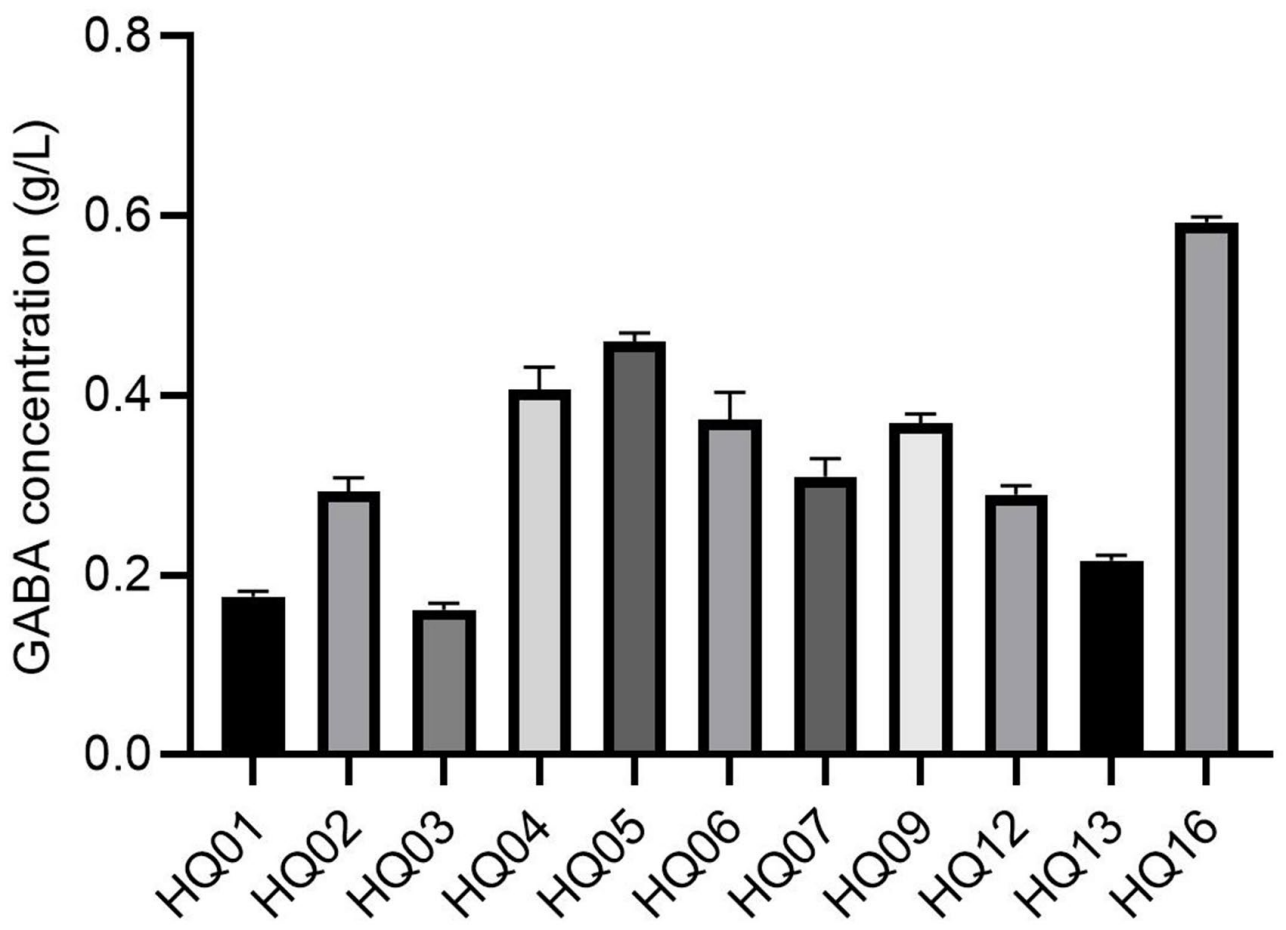
GABA production of 11 *L. plantarum* strains. All results are represented as the mean ± SD. GABA indicates gamma-aminobutyric acid.

The ability of the potential probiotic strains to produce GABA was evaluated in our study. As shown in Figure 2, all 11 *L. plantarum* strains produced GABA. However, the extracellular concentration of GABA differed among the 11 *L. plantarum* strains and ranged from 0.16 g/L (HQ03) to 0.60 g/L (HQ16). The highest GABA producers (>0.4 g/L) were strains HQ04, HQ05, and HQ16. The lowest GABA levels (<0.2 g/L) were observed for strains HQ01 and HQ03.

#### 3.3.3 Autoaggregation and coaggregation ability

The auto- and coaggregation abilities of the 11 *L. plantarum* strains are summarized in Table 1. Furthermore, the autoaggregation abilities of the 11 *L. plantarum* strains ranged from 8.66 ± 0.61% to 72.72 ± 1.31%. The highest autoaggregation abilities were observed in strains HQ03 (72.72 ± 1.31%), HQ04 (72.09 ± 1.39%), and HQ13 (66.44 ± 1.73%). All examined *L. plantarum* strains co-aggregated with *E. coli* CVCC196 and *S. aureus* BJ216. *L. plantarum* strains HQ01, HQ03, HQ04, HQ05, and HQ13 showed the strongest degree of coaggregation after 5 h of incubation. Specifically, the observed values for coaggregation of HQ03 with *E. coli* CVCC196 and *S. aureus* BJ216 were 80.05 ± 1.04% and 21.50 ± 0.58%, respectively, whereas those for HQ04 were 76.52 ± 1.89% and 33.51 ± 0.58%, respectively.

**Table 1:** Autoaggregation and coaggregation ability of 11 *L. plantarum* strains.

Strain	Autoaggregation (%)	Coaggregation (%)	
		*E. coli* CVCC196	*S. aureus* BJ216
HQ01	23.01 ± 0.91	21.72 ± 0.57	27.10 ± 0.75
HQ02	8.66 ± 0.61	1.21 ± 0.13	3.24 ± 0.16
HQ03	72.72 ± 1.31	80.05 ± 1.04	21.50 ± 0.58
HQ04	72.09 ± 1.39	76.52 ± 1.89	33.51 ± 0.58
HQ05	21.43 ± 0.29	25.74 ± 0.68	13.51 ± 0.55
HQ06	23.71 ± 0.24	24.57 ± 1.55	10.46 ± 0.40
HQ07	15.83 ± 0.30	7.72 ± 0.61	11.83 ± 0.75
HQ09	12.91 ± 0.72	12.47 ± 1.31	22.10 ± 0.39
HQ12	27.90 ± 0.88	19.18 ± 0.88	1.31 ± 0.39
HQ13	66.44 ± 1.73	29.83 ± 1.37	17.76 ± 1.17
HQ16	13.96 ± 1.00	13.87 ± 0.74	13.67 ± 1.31

All results are represented as mean ± SD.

#### 3.3.4 Antibacterial activity

Table 2 presents the inhibitory ability of the 11 *L. plantarum* strains against the pathogenic indicator bacteria (*E. coli* CVCC196 and *S. aureus* BJ216). Strains HQ01, HQ02, HQ03, HQ05, HQ12, HQ13, and HQ16 did not form inhibitory areas around *E. coli* CVCC196. However, the strains HQ04, HQ06, HQ07, and HQ09 exhibited excellent bacteriostatic properties against *E. coli* CVCC196. Furthermore, strains HQ01, HQ02, HQ03, HQ04, HQ05, and HQ09 formed > 12 mm inhibition zones against *S. aureus* BJ216. The inhibition zones observed for strains HQ07, HQ12, and HQ13 against *S. aureus* BJ216 were >10 mm. These results indicated that the strains HQ04, HQ07, and HQ09 elicited the strongest inhibitory effects on pathogenic bacteria among the isolates.

**Table 2:** Diameter of inhibition zones of 11 *L. plantarum* strains from kimchi against *E. coli* CVCC196 and *S. aureus* BJ216.

Strain	*E. coli* CVCC196			*S. aureus* BJ216		
	CFS	CFS (1 mol/mL NaOH treatment)	CFS (trypsin treatment)	CFS	CFS (1 mol/mL NaOH treatment)	CFS (trypsin treatment)
HQ01	0.00 ± 0.00	—	—	13.07 ± 0.31	0.00 ± 0.00^**^	10.43 ± 0.12^**^
HQ02	0.00 ± 0.00	—	—	14.00 ± 0.20	0.00 ± 0.00^**^	10.07 ± 0.23^**^
HQ03	0.00 ± 0.00	—	—	14.60 ± 0.40	0.00 ± 0.00^**^	0.00 ± 0.00^**^
HQ04	13.27 ± 0.31	0.00 ± 0.00^**^	0.00 ± 0.00^**^	14.67 ± 0.12	0.00 ± 0.00^**^	0.00 ± 0.00^**^
HQ05	0.00 ± 0.00	—	—	12.27 ± 0.31	0.00 ± 0.00^**^	0.00 ± 0.00^**^
HQ06	10.47 ± 0.61	0.00 ± 0.00^**^	0.00 ± 0.00^**^	0.00 ± 0.00	—	—
HQ07	9.80 ± 0.20	0.00 ± 0.00^**^	10.06 ± 0.15	11.73 ± 0.31	0.00 ± 0.00^**^	0.00 ± 0.00^**^
HQ09	12.20 ± 0.40	0.00 ± 0.00^**^	9.07 ± 0.15^*^	14.87 ± 0.23	0.00 ± 0.00^**^	0.00 ± 0.00^**^
HQ12	0.00 ± 0.00	—	—	10.33 ± 0.23	0.00 ± 0.00^**^	11.07 ± 0.15^*^
HQ13	0.00 ± 0.00	—	—	10.6 ± 0.40	0.00 ± 0.00^**^	10.17 ± 0.15
HQ16	0.00 ± 0.00	—	—	8.67 ± 0.31	0.00 ± 0.00^**^	0.00 ± 0.00^**^

*Indicates a significant difference between treatments (*p* < 0.05) obtained using ANOVA. The representation of *p*-values is as follows: “*” for *p* < 0.05, “**” for *p* < 0.01. “—”Indicates no results. CFS indicates cell-free supernatant; all results are represented as mean ± SD.

### 3.4 Complete genome information of the *Lactiplantibacillus plantarum* HQ04 strain

**Figure 3:**
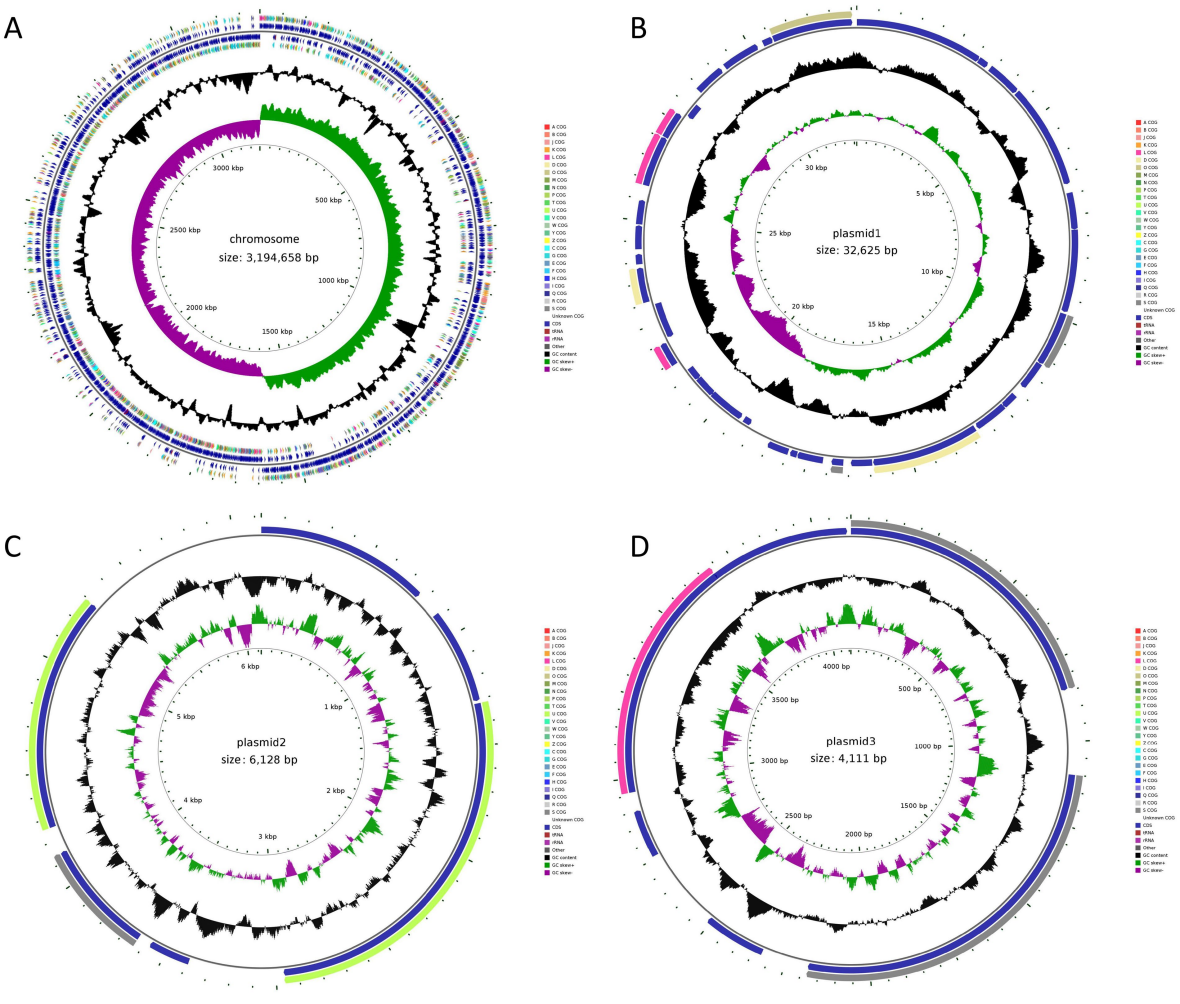
Genome circle diagram of *L. plantarum* HQ04 strain. **(A–D)** Represent the genomic map of chromosomal and plasmid1, plasmid2, and plasmid3, respectively.

Based on the probiotic properties of the 11 *L. plantarum* strains, HQ04 was selected as the most effective strain and further assessed using whole genome sequencing analysis. The initial total genomic DNA sequencing was performed using Illumina NovaSeq PE150 and Oxford Nanopore ONT sequencing platforms, which yielded four circular units, one large circular 3,194,658 bp chromosome, and three plasmids that were 32,625, 6,128, and 4,111 bp long. The respective guanine and cytosine (G + C) contents were 44.62, 36.21, 40.75, and 38.75%, which were similar to those of other *L. plantarum* strains. The HQ04 strain chromosomal and plasmid genome circle diagrams are shown in Figure 3. The HQ04 strain chromosome contained 3,000 open reading frames (ORFs), which accounted for 93.9% of the total length. Additionally, it contained 65 tRNAs, 16 rRNAs, and 59 ncRNAs. The 3 plasmids contained 33, 6, and 6 ORFs, respectively (Supplementary Table S2).

**Figure 4:**
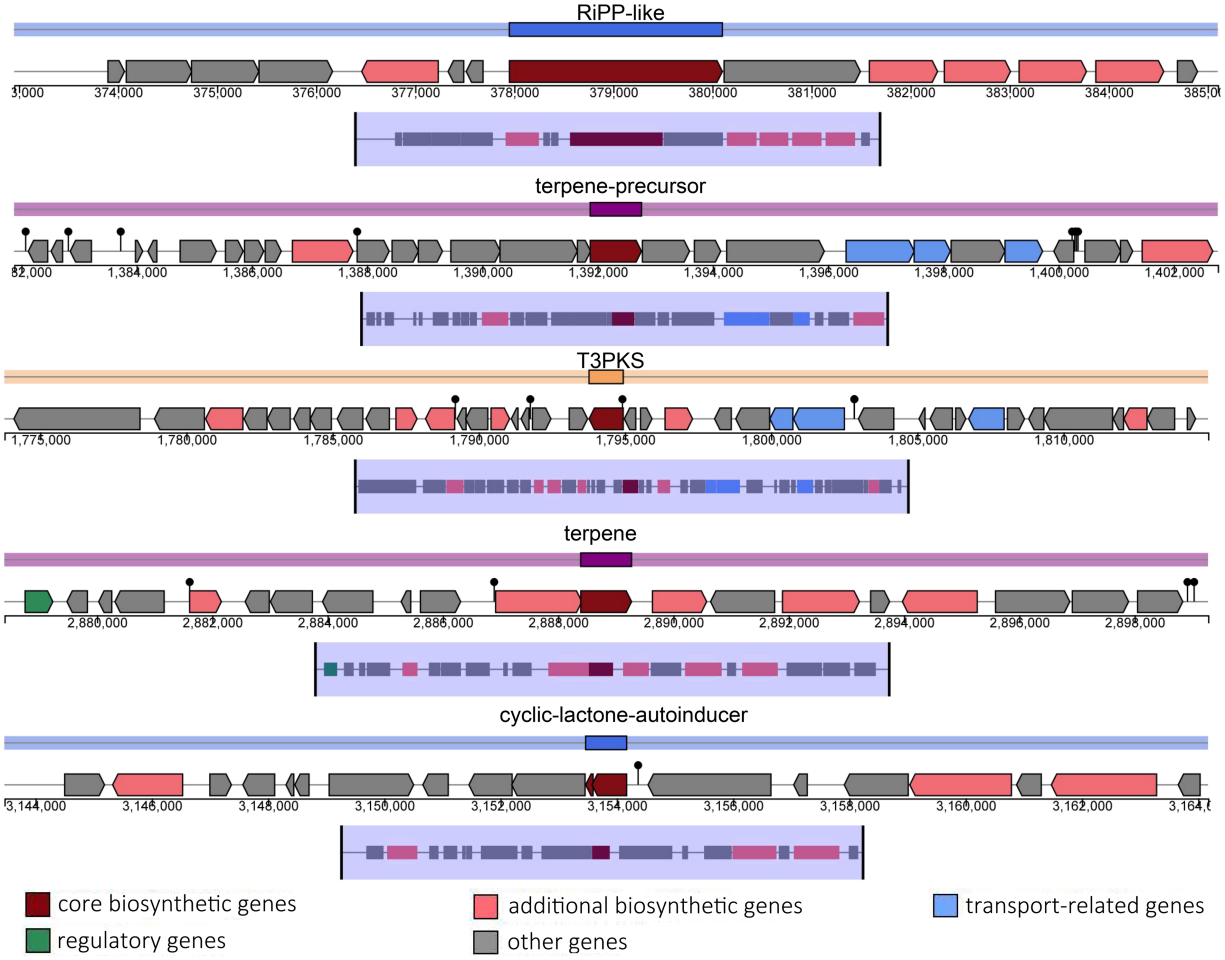
Genomic maps of the clusters of RiPP-like, terpene-precursor, T3PKS, terpene, and cyclic-lactone-autoinducer regions of *L. plantarum* HQ04. Gene clusters are represented by arrows with different colors corresponding to operons of different functions. RiPP indicates ribosomally synthesized and post-translationally modified peptides; T3PKS indicates type III polyketide synthase.

**Figure 5:**
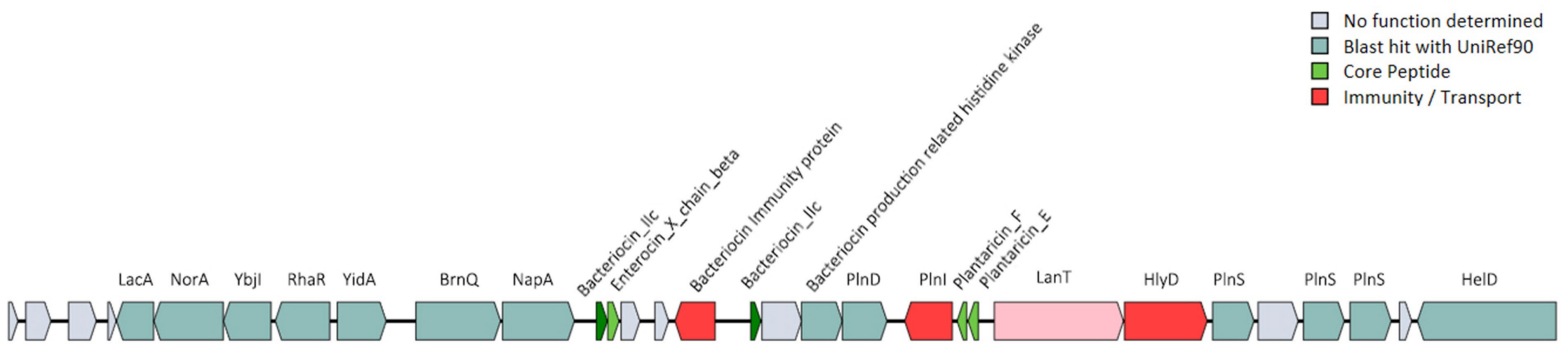
Predicted bacteriocin-encoding genes in the *L. plantarum* HQ04 genome. The gene clusters encoding bacteriocins are represented by arrows with different colors corresponding to the operons of different functions.

The probiotic properties of the HQ04 strain were confirmed *in vitro*, and genome analysis supported some of the potential probiotic properties of this strain. The HQ04 genome was searched for multiple gene clusters related to the synthesis of antibacterial metabolites using the antiSMASH tool (Figure 4 and Supplementary Table S3), and five multiple gene clusters were identified to putatively encode the synthesis of antimicrobial compounds, including clusters for the synthesis of ribosomally synthesized and post-translationally modified peptides (RiPP)-like, terpene-precursor, Type III polyketide synthase (T3PKS), terpene, and cyclic-lactone-autoinducer. In addition, a bacteriocin-encoding gene cluster was identified in the HQ04 genome using BAGEL4, which contained three hot spots, and we speculated that it could synthesize Bacteriocin IIc, Enterocin X chain beta, and Plantaricin E/F. Additionally, bacteriocin immunity protein, bacteriocin production-related histidine kinase, immunity protein PlnI, ABC transporter PlnH (LanT), accessory factor for ABC transporter PlnH (HlyD), and PlnS were identified (Figure 5).

## 4 Discussion

### 4.1 Bacterial diversity and community structure of kimchi samples

High-throughput sequencing technology has been widely and effectively applied to the study of complex microorganisms in fermented foods (Thierry et al., 2023; Feng et al., 2025). Hence, we performed 16S rRNA gene amplicon community profiling to understand the bacterial diversity in kimchi samples from 10 districts in Yibin City. To the best of our knowledge, this study is the first to characterize the microbial community in homemade fermented kimchi from Yibin City, which is the first city along the Yangtze River and is well-known for its diverse range of traditionally fermented foods. The results showed discrepancies in the relative abundances of the top nine dominant phyla in the bacterial community. Firmicutes was the predominant phylum in the kimchi samples, and Lactobacillales was the dominant taxon at the order level. Members of Lactobacillales belong to the phylum Firmicutes, which plays the most significant role in shaping the kimchi microenvironment (Thierry et al., 2023; Liu and Tong, 2017). Additionally, *Levilactobacillus* and *Lactiplantibacillus* appeared to be the primary Lactobacillales, and this finding is consistent with that of another study that focused on the microbiota of homemade fermented kimchi in Western countries (Thierry et al., 2023). These results are corroborated by the findings of studies involving bacterial cultures. For example, Abouloifa et al. (2020) isolated two *Lactiplantibacillus* strains and one *Levilactobacillus* strain from traditionally fermented olives and verified that these strains possessed the ability to produce organic acids, fructo-oligosaccharides, and xylo-oligosaccharides, which are involved in the formation of the flavor of fermented foods. Similarly, *Levilactobacillus* and *Lactiplantibacillus* have been cited as the most frequently isolated LAB species from homemade Chinese kimchi, and these strains are strongly linked to kimchi flavor, which reinforces the inference that these genera are key players in spontaneously fermented kimchi (Liu et al., 2024). Additionally, *Pediococcus* and *Lactobacillus*, which are also of high significance in fermented food, were present at a lower level in the kimchi samples (Zhao et al., 2023). However, the low abundance of potential pathogen-containing genera such as *Acinetobacter*, *Pseudomonas*, *Klebsiella*, and *Serratia* in some samples, albeit at low abundances, highlights the importance of screening for safe and functional probiotics in these complex ecosystems.

Chao1 and Shannon indices indicate community richness and evenness, respectively. Compared with the Chao1 and Shannon indices of other samples (soil, feces, and sewage) enriched with microorganisms (Galic et al., 2024), the diversity of the bacterial community in the kimchi samples was relatively low. This may be attributed to the selective pressure of the kimchi environment, which is characterized by low pH, high salinity, and anaerobic conditions (Thierry et al., 2023). Similar results have been reported for other types of fermented kimchi (Feng et al., 2025; Liu and Tong, 2017; Liu et al., 2019). Beta diversity analysis showed that the microbial communities did not cluster according to their geographical districts of origin. This lack of geographical patterning has also been previously observed in a study of Korean household kimchi (Lee et al., 2017). In that study, high-throughput sequencing was performed to analyze 69 private kimchi samples from households. The results indicated that homemade fermented kimchi may be regarded as an individualized bacterial ecosystem. This implies that each homemade kimchi sample from Yibin can be regarded as an independent bacterial ecosystem that represents a vast and largely untapped reservoir of novel microbial resources for probiotic screening.

### 4.2 Assessment of probiotic properties

Based on the results of morphological and molecular biology-based analyses, 11 *L. plantarum* strains were isolated from kimchi samples. *L. plantarum* is one of the most versatile and commonly found LAB strains in fermented foods (Zhang et al., 2023). It is an economically important starter culture bacterium that initiates food fermentation, and certain strains are sold as probiotics (Brooijmans et al., 2009). Therefore, these 11 *L. plantarum* strains warrant further analysis for their probiotic properties. The results of the acid and bile salt tolerance tests indicated that the *L. plantarum* HQ04 and HQ06 strains exhibited notable acid and salt resistance. The survival of *L. plantarum* strains isolated from fermented foods in acidic and bile salt environments has been reported previously (Iarusso et al., 2025). This reflects the ability of potential probiotic strains to tolerate intestinal acid and bile salts and is of immense importance for their survival and growth in the gastrointestinal tract, which is a major requirement for probiotic selection (Reuben et al., 2019). In the present study, *L. plantarum* HQ04, *L. plantarum* HQ05, and *L. plantarum* HQ16 strains showed efficient GABA production (0.4 to 0.59 g/L), which was higher than that of *L. plantarum* 8,014 (0.16 g/L) reported by Li et al. (2020). As a LAB bioactive metabolite, GABA is an important inhibitory neurotransmitter in the central nervous system of mammals (Wang et al., 2023). The multiple physiological functions of GABA-producing LAB, such as increasing appetite, promoting digestion, treating epilepsy, inhibiting cancer cell proliferation, and boosting immunity, have been studied intensively (Iorizzo et al., 2023). These characteristics laid the foundation for further analysis.

Probiotics effectively colonize the host intestinal tract and prevent pathogenic bacteria from colonizing the intestine. Autoaggregation and coaggregation are important indicators for evaluating adherence ability *in vitro* (Wang et al., 2023; Elnar and Kim, 2025). In this study, three strains (*L. plantarum* HQ03, HQ04, and HQ13) showed good autoaggregation and coaggregation abilities. The observed values were higher than those reported by Lin et al. (2020), who reported autoaggregation and coaggregation for *L. plantarum* strains in the range of 25.74–30.10% and 13.36–15.23%, respectively. These results indicate that *L. plantarum* HQ03, *L. plantarum* HQ04, and *L. plantarum* HQ13 can adhere, colonize, and survive well in the gastrointestinal tract and potentially inhibit infection by some pathogenic bacteria.

Antimicrobial activity is one of the most important criteria for selecting new probiotic strains (Jiang et al., 2023). Antimicrobial activity is related to the production of bacteriocins, organic acids, alcohols, hydrogen peroxide, and antimicrobial peptides, which play essential protective roles against pathogens (Huidrom et al., 2024; Wang et al., 2023). As shown in Table 1, the HQ04 strain completely lost its inhibitory effect on *E. coli* CVCC196 and *S. aureus* BJ216 after the pH of the CFS was adjusted to 7.0 and trypsin treatment. This suggests that the antibacterial effect of the HQ04 strain may be attributed to its production of organic acids and bacteriocins. Mechanistically, the organic acids produced by *L. plantarum* disrupt the proton motive force of pathogenic bacteria, which leads to intracellular acidification and loss of viability (Thierry et al., 2015). The proteinaceous nature of these antimicrobial compounds was confirmed by trypsin sensitivity analysis. The results supported the role of bacteriocins in the observed antibacterial activity. Additionally, the inhibitory effect of HQ07 and HQ09 strains on *E. coli* CVCC196 was significantly influenced by changes in pH. Among the 11 *L. plantarum* strains, HQ04, HQ07, and HQ09 showed the largest zones of inhibition against pathogens, which indicates that these strains elicit excellent antibacterial activity. Bacteriocins are naturally occurring antibacterial compounds produced by most bacterial species and are widely involved in the antibacterial effects of probiotics; however, most of these compounds have not been identified (Yang et al., 2014; Reuben et al., 2019). Therefore, further studies are required to investigate the antibacterial mechanisms of the HQ04 strain.

### 4.3 Complete genome information of *Lactiplantibacillus plantarum* HQ04 strain

The complete genome sequence of the most promising strain, *L. plantarum* HQ04, provided molecular insights into its probiotic properties. Three plasmids were identified in *L. plantarum* HQ04. *L. plantarum* is one of the most versatile species and usually carries different plasmids. Crowley et al. (2013) identified 10 plasmids (pLp16A-pLp16L) in *L. plantarum* strain 16. Nevertheless, obtaining the complete genome of many *L. plantarum* strains has proven difficult because of the large plasmid complement (Chen et al., 2014). McLeod et al. (2019) have reported that the ONT MinION sequencer was particularly useful in resolving the unusually large number of plasmids in an *L. plantarum* strain. The ONT MinION sequencer used a combination of Illumina MiSeq and ONT MinION to obtain the complete genome of the *L. plantarum* MF1298 strain, which contained 14 plasmids. In this study, we obtained the complete genome of the *L. plantarum* HQ04 strain using the Illumina NovaSeq PE150 and Oxford Nanopore ONT sequencing platforms, which effectively ensured the integrity and accuracy of the HQ04 genome.

In total, the *L. plantarum* HQ04 genome comprised five secondary metabolite gene clusters, including RiPP-like, terpene-precursor, T3PKS, terpene, and cyclic lactone autoinducer. This was consistent with the cyclic lactone autoinducer, terpenes, T3PKS, and RiPP-like gene clusters observed in the genome of the *L. plantarum* 13-3 strain, which is a food fermentation strain isolated from Tibetan kefir grains (Aziz et al., 2022). These compounds play crucial roles in antibacterial, antifungal, anticancer, immunoregulatory, and other biological processes (Naughton et al., 2017; Glassey et al., 2025; Iliev et al., 2025). This implies that *L. plantarum* HQ04 is capable of producing a variety of antibacterial compounds that not only possess antimicrobial properties but also play critical roles in shaping the microbiota by inhibiting pathogens.

Most importantly, a bacteriocin-encoding gene cluster was identified in the *L. plantarum* HQ04 genome using BAGEL4, and several genes (*plnI*, *lanT*, *hlyD*, and *plnS*) in the cluster are related to the synthesis of antimicrobial compounds (Rizzello et al., 2014). Notably, the gene cluster encodes the core peptides of Bacteriocin IIc, Enterocin X chain beta, and Plantaricin E/F. Enterocin X is a novel class IIb bacteriocin that was first identified in *Enterococcus faecium* KU-B5. It comprises two antimicrobial peptides (Xalpha and Xbeta), and the complementarity of Xalpha and Xbeta enhances the antibacterial activity (Hu et al., 2010). Plantaricin E/F is a two-peptide class IIb bacteriocin that was first discovered in *L. plantarum* C11. Anderssen et al. (1998) showed that Plantaricin E/F kills microbial cells by permeabilizing the cell membranes. Additionally, plantaricin E/F structures have been discovered in *L. plantarum* strain DHCU70, *L. plantarum* strain DKP1, and two strains showing bacteriocin production isolated from Indian fermented foods (Goel et al., 2020). Huidrom et al. (2024) found Enterocin X and Plantaricin E/F in the genome of the *L. plantarum* BRD3A strain isolated from Atingba, which is a traditional fermented rice-based beverage. Bacteriocin IIc (class IIc) is an important member of class II bacteriocins and belongs to the non-pediocin one-peptide circular bacteriocin subgroup. It is highly effective against methicillin-resistant *S. aureus* (Mathur et al., 2017). Class II bacteriocins (Plantaricin E/F, Enterocin X) primarily exert their antibacterial effects through membrane disruption, whereas class IIb bacteriocins act synergistically to form pores in the cytoplasmic membrane of target bacteria, which leads to ion leakage, collapse of the proton motive force, and eventually cell death (Nissen-Meyer et al., 2009). Class IIc bacteriocins often adopt a circular structure that enhances their stability and facilitates their insertion into bacterial membranes, which causes depolarization and content leakage (Mathur et al., 2017). Currently, class II bacteriocins and the strains that produce them are increasingly being used to prevent food spoilage and pathogenic bacterial growth in the food industry (Wang et al., 2022). In the present study, the presence of genetic material of both class IIb and IIc bacteriocins in HQ04 suggests a complementary and potentially broad-spectrum antibacterial mechanism that is effective against pathogens. Thus, the *L. plantarum* HQ04 strain might be a potential probiotic strain with applications in the food industry. In the context of the current study, the antibacterial properties of *L. plantarum* HQ04 were attributed to bacteriocins, based on genomic analysis and crude enzyme treatment. However, the active bacteriocin was not purified, and its specific underlying inhibitory mechanisms need to be characterized. In future studies, we aim to validate the safety, probiotic functions, and antimicrobial mechanisms of the *L. plantarum* HQ04 strain and bacteriocins using *in vivo* animal experiments.

## 5 Conclusion

In conclusion, our study provides the first comprehensive analysis of the bacterial community structure in homemade kimchi produced in Yibin City. *Levilactobacillus* and *Lactiplantibacillus* were the major microorganisms identified at the genus level in kimchi samples. As the microbial communities in each sample formed an independent bacterial ecosystem, homemade fermented kimchi could be an important source for screening probiotics. Furthermore, we successfully isolated and characterized 11 *L. plantarum* strains. Among these, the *L. plantarum* HQ04 strain showed probiotic properties. Notably, five secondary metabolite gene clusters were detected, and a bacteriocin-encoding gene cluster was found in the *L. plantarum* HQ04 genome. Notably, the genetic presence of both class IIb and IIc bacteriocins in the *L. plantarum* HQ04 genome suggests a complementary and potentially broad-spectrum antibacterial mechanism that is effective against pathogens. This study showed that microbial community composition differed in various homemade fermented kimchi samples from Yibin and that the *L. plantarum* HQ04 strain is a potential probiotic candidate for use in the food and healthcare industries. Future studies should explore the *in vivo* safety, probiotic efficacy, and antimicrobial mechanisms of *L. plantarum* HQ04 and its bacteriocins.

## Data Availability

The raw data were uploaded to the NCBI Sequence Read Archive (SRA) under accession number PRJNA1309784. The 11 *L. plantarum* strains 16S rRNA sequences have been deposited in the NCBI GenBank under accession numbers PV876049–PV876059. The *L. plantarum* HQ04 chromosomes and plasmids sequences were also uploaded to the NCBI GenBank under accession numbers JBQRVB010000001–JBQRVB010000004.

## Footnotes

1
https://qiime2.org/

2
https://antismash.secondarymetabolites.org/#!/start

3
http://bagel.molgenrug.nl/
