# Supplementary material for: Bacterial community in homemade kimchi and probiotic characterization of Lactiplantibacillus plantarum HQ04
Source: Front Microbiol. 2025 Nov 28;16:1700442. doi: 10.3389/fmicb.2025.1700442 (PMC12698544; doi:10.3389/fmicb.2025.1700442)
Supplement: Supplementary file 1 [file Supplementary_file_1.docx]

Supplementary Material

# Supplementary Figures and Tables

##
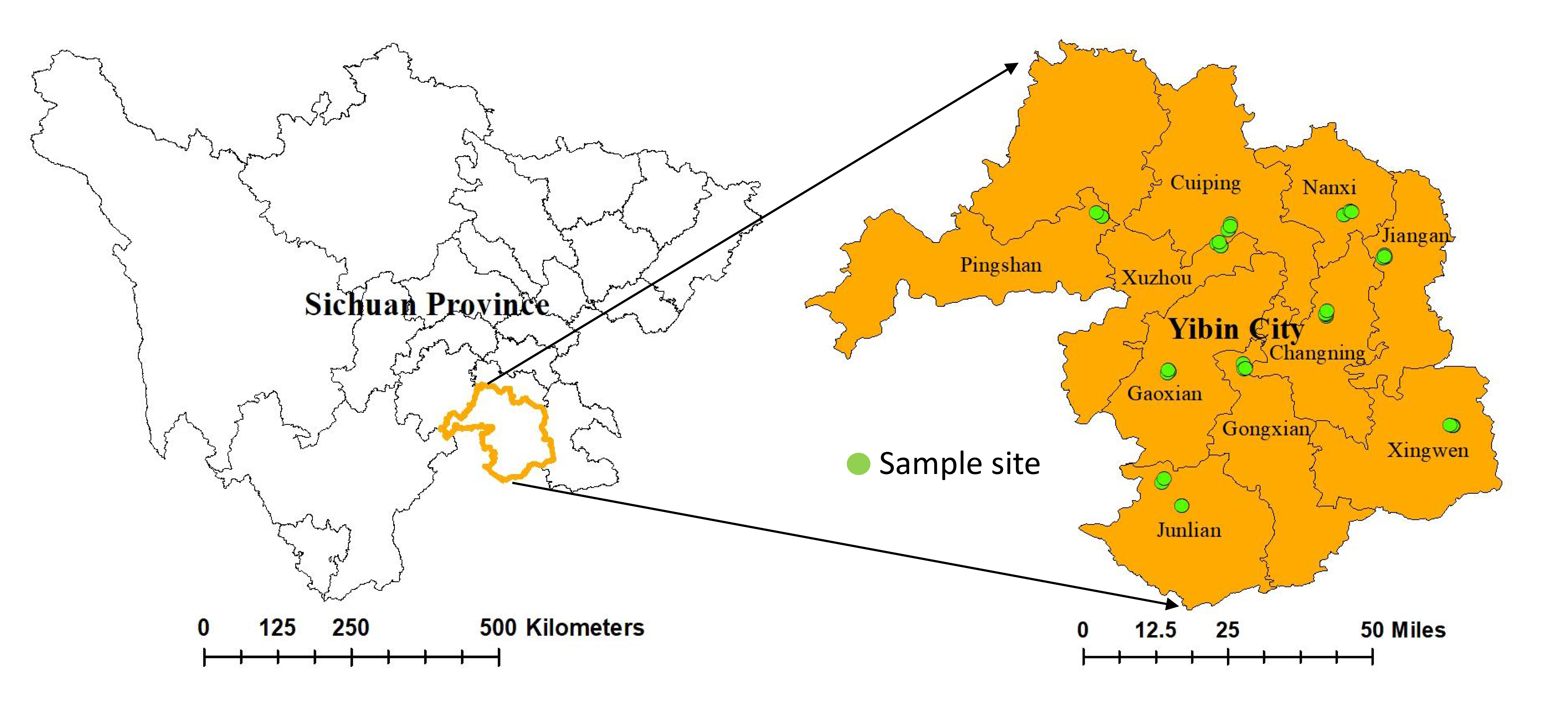
Supplementary Figures

**Supplementary Figure 1.** Sampling sites in the ten district Yibin City, Sichuan province, China.


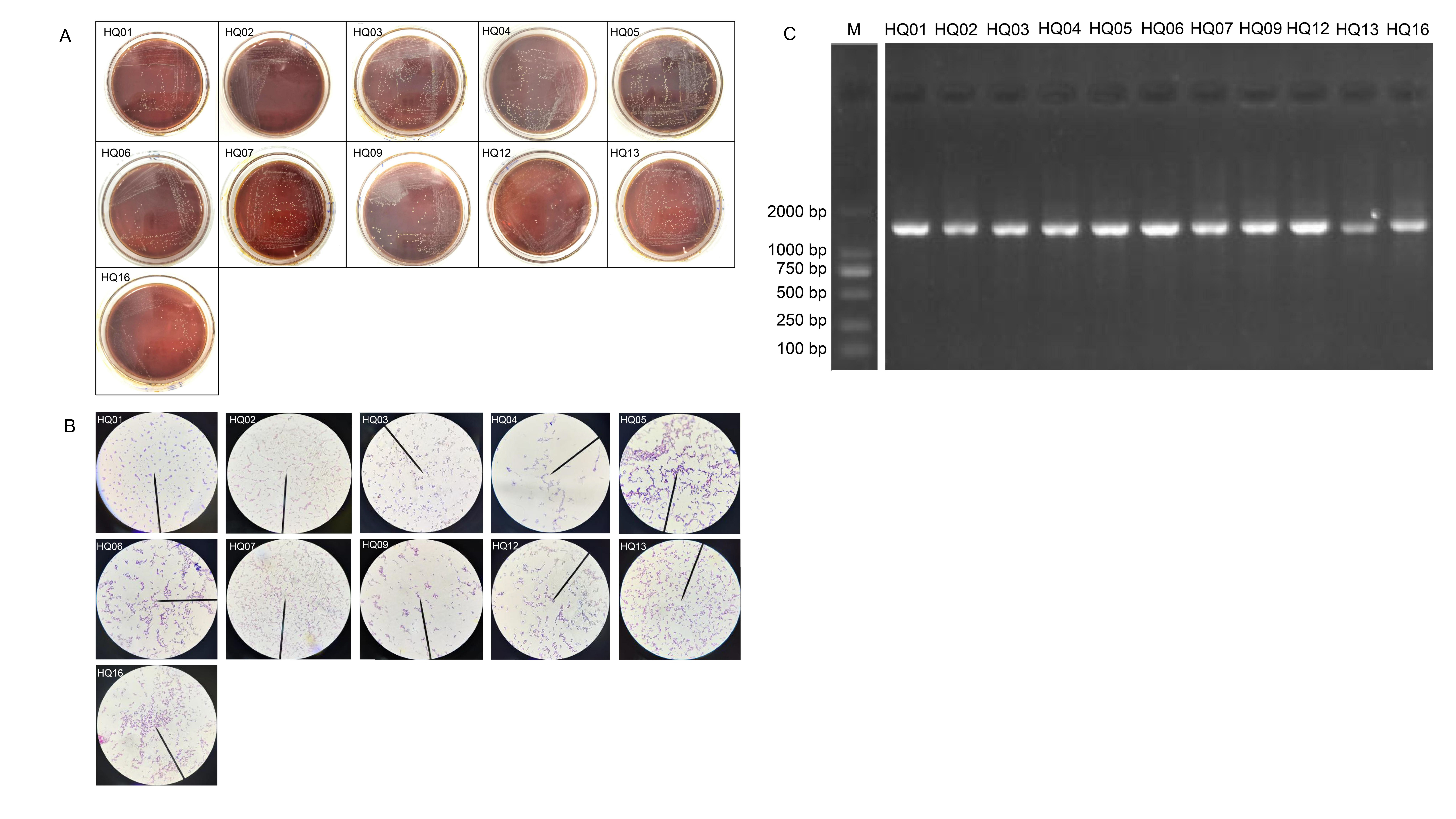


**Supplementary Figure** **S2.** The results of isolation and identification of 11 *L. plantarum* strains in kimchi. (A) The colony morphology on the MRS medium; (B) The result of gram staining; (C) Agarose gel electrophoresis of PCR products after amplification of 16S rRNA gene.

## Supplementary Tables

**Supplementary Table S1.** Survival rate (%) of 11 *L. plantarum* strains after exposure to acid and bile salt

| Strain | pH 2 | pH 3 | pH 4 | 0.1%  bile salt | 0.2%  bile salt | 0.3%  bile salt |
| --- | --- | --- | --- | --- | --- | --- |
| HQ01 | 58.13±2.10 | 58.81±2.24 | 78.59±1.19 | 21.53±0.16 | 2.24±0.04 | 2.21±0.03 |
| HQ02 | 27.57±1.77 | 98.66±0.52 | 94.83±0.08 | 49.65±0.30 | 8.24±0.03 | 0.85±0.06 |
| HQ03 | 56.64±0.38 | 66.94±0.27 | 81.03±0.25 | 23.83±1.33 | 4.68±0.09 | 1.35±0.02 |
| HQ04 | 57.20±1.25 | 82.65±0.48 | 89.65±0.89 | 46.62±0.91 | 14.58±0.09 | 9.33±0.00 |
| HQ05 | 52.41±0.90 | 77.87±0.53 | 88.53±0.53 | 6.43±0.05 | 7.41±0.18 | 0.35±0.03 |
| HQ06 | 71.37±0.64 | 80.90±1.03 | 87.29±1.32 | 31.56±2.01 | 18.84±1.27 | 11.32±0.05 |
| HQ07 | 68.40±4.94 | 74.15±0.88 | 98.67±0.65 | 14.44±1.84 | 1.06±0.01 | 0.63±0.03 |
| HQ09 | 16.72±0.55 | 33.79±0.86 | 84.47±0.26 | 0.02±0.02 | 0.01±0.00 | 0.01±0.00 |
| HQ12 | 75.36±0.54 | 85.14±0.73 | 94.55±0.56 | 78.49±2.85 | 9.42±0.61 | 1.75±0.16 |
| HQ13 | 45.38±1.04 | 90.22±2.20 | 85.24±4.21 | 8.76±0.08 | 0.15±0.01 | 0.05±0.01 |
| HQ16 | 14.89±0.86 | 69.14±3.40 | 60.62±3.69 | 25.43±0.12 | 7.55±0.69 | 1.07±0.06 |

All results are represented as mean ± SD.

**Supplementary Table S2.** Genome assembly and annotation of *L. plantarum* HQ04 strain.

| Type | Name | Size (bp) | GC% | ORF | rRNA | tRNA | ncRNA |
| --- | --- | --- | --- | --- | --- | --- | --- |
| Chromosome | HQ04 | 3,194,658 | 44.62 | 3,000 | 16 | 65 | 59 |
| Plasmid1 | pHQ04-1 | 32,625 | 36.21 | 33 | 0 | 0 | 0 |
| Plasmid2 | pHQ04-2 | 6,128 | 40.75 | 6 | 0 | 0 | 0 |
| Plasmid3 | pHQ04-3 | 4,111 | 38.75 | 6 | 0 | 0 | 1 |

**Supplementary Table S3.** Predicted secondary metabolites with antibacterial activity identified in the genome of *L. plantarum* strain HQ04.

| Cluster | Genome location | Closest match in  NCBI  (accession no.) | Identity  (%) | Product blast in NCBI |
| --- | --- | --- | --- | --- |
| RiPP-like | 372951-  385102 | *L. plantarum* strain LP01 (CP170480.1) | 99.95 | Bacteriocin immunity protein, sensor histidine kinase, lysostaphin resistance A-like protein, two-peptide bacteriocin plantaricin EF subunit PlnF, two-peptide bacteriocin plantaricin EF subunit PlnE, peptide cleavage/export ABC transporter, HlyD family secretion protein, CPBP family intramembrane glutamic endopeptidase, lysostaphin resistance A-like protein, RNA polymerase recycling motor HelD |
| terpene-  precursor | 1381875-1402765 | *L. plantarum* strain SCB0151 (CP084715.1) | 99.97 | dUTPase, TetR/AcrR family transcriptional regulator, 50S ribosomal protein L21, ribosomal-processing cysteine protease Prp, 50S ribosomal protein L27, aminopeptidase P family protein, elongation factor P, Asp23/Gls24 family envelope stress response protein, transcription antitermination factor NusB, methenyltetrahydrofolate cyclohydrolase, exodeoxyribonuclease VII large subunit, polyprenyl synthetase family protein, TlyA family rRNA (cytidine-2'-O)-methyltransferase, arginine repressor, DNA repair protein RecN, betaine/proline/choline family ABC transporter ATP-binding protein, ABC transporter permease, osmoprotectant ABC transporter substrate-binding protein, ABC transporter permease, guanylate kinase, DNA-directed RNA polymerase subunit omega, bifunctional phosphopantothenoylcysteine decarboxylase/phosphopantothenate--cysteine ligase CoaBC, primosomal protein N |
| T3PKS | 1773777-1814946 | *L. plantarum* strain MD159 (CP166737.1) | 99.94 | ribosome maturation factor RimP, DNA polymerase III subunit alpha, proline--tRNA ligase, RIP metalloprotease RseP, phosphatidate cytidylyltransferase, isoprenyl transferase, ribosome recycling factor, UMP kinase, translation elongation factor Ts, 30S ribosomal protein S2, HAD family hydrolase, D-2-hydroxyacid dehydrogenase, GIY-YIG nuclease family protein, tRNA1(Val) (adenine(37)-N6)-methyltransferase, lysophospholipid acyltransferase family protein, YneF family protein, DUF896 domain-containing protein, transcriptional repressor LexA, DNA alkylation repair protein, hydroxymethylglutaryl-CoA synthase, glycosyltransferase family 2 protein, helix-turn-helix domain containing protein, LCP family protein, ABC transporter ATP-binding protein, ABC transporter permease subunit, FtsW/RodA/SpoVE family cell cycle protein, class I SAM-dependent methyltransferase, heavy metal-binding domain-containing protein, MFS transporter, helix-turn-helix domain-containing protein, adenine phosphoribosyltransferase, single-stranded-DNA-specific exonuclease RecJ, lipopolysaccharide assembly LapA domain-containing protein, SDR family NAD(P)-dependent oxidoreductase, ribonuclease Z, GTPase ObgE |
| [terpene](https://docs.antismash.secondarymetabolites.org/glossary/#terpene) | 2878395-2899276 | *L. plantarum* strain CGMCC 14177 (CP143285.1) | 99.96 | Fur family transcriptional regulator, bacteriocin immunity protein, PLDc N-terminal domain-containing protein, metallophosphoesterase, GNAT family protein, CidA/LrgA family protein, LrgB family protein, DegV family protein, zinc metallopeptidase, phytoene desaturase family protein, phytoene/squalene synthase family protein, alpha/beta hydrolase, glycerate kinase, NAD(P)/FAD-dependent oxidoreductase, adenylosuccinate lyase, adenylosuccinate synthase, GMP reductase, tyrosine-protein phosphatase, cardiolipin synthase, |
| cyclic-lactone-autoinducer | 3143466-3164171 | *L. plantarum* strain K25 (CP020093.1) | 99.98% | GntR family transcriptional regulator, N-acetylmannosamine-6-phosphate 2-epimerase, FAD-dependent oxidoreductase, TIGR02328 family protein, folate family ECF transporter S component, GlsB/YeaQ/YmgE family stress response membrane protein, catalase, Spx/MgsR family RNA polymerase-binding regulatory protein, LytTR family DNA-binding domain-containing protein, GHKL domain-containing protein, cyclic lactone autoinducer peptide, accessory gene regulator AgrB, ATP-dependent protease ClpL, lactate oxidase, thiamine pyrophosphate-dependent enzyme, Rrf2 family transcriptional regulator, pyruvate oxidase, PTS glucitol/sorbitol transporter subunit IIA, PTS glucitol/sorbitol transporter subunit IIB |
